# Evidence for implementation of interventions to promote mental health in the workplace: a systematic scoping review protocol

**DOI:** 10.1186/s13643-020-01570-9

**Published:** 2021-01-28

**Authors:** Charlotte Paterson, Caleb Leduc, Margaret Maxwell, Birgit Aust, Benedikt L. Amann, Arlinda Cerga-Pashoja, Evelien Coppens, Chrisje Couwenbergh, Cliodhna O’Connor, Ella Arensman, Birgit A. Greiner

**Affiliations:** 1 NMAHP-RU, University of Stirling,; 2 School of Public Health, University College Cork, Cork, Ireland; 3 National Suicide Research Foundation, Cork, Ireland; 4 National Research Centre for the Working Environment, Copenhagen, Denmark; 5 Centro Forum Research Unit, Institut de Neuropsiquiatria i Addicions (INAD), Parc de Salut Mar, Hospital del Mar Medical Research Institute (IMIM), Autonomous University Barcelona, CIBERSAM, Barcelona, Spain; 6 London School of Hygiene and Tropical Medicine, London, UK; 7 Centre for Health Research and Consultancy, Katholieke Universiteit Leuven, Leuven, Belgium; 8 Kenniscentrum Phrenos, Utrecht, Netherlands; 9 Australian Institute for Research, Griffith University, Mount Gravatt, Australia; 10 International Association for Suicide Prevention (IASP), Washington, DC USA

**Keywords:** Barriers and facilitators, RE-AIM, Workplace, Mental health promotion, Implementation science, Scoping review, Organisational interventions, Workplace interventions, Process evaluation, Wellbeing promotion

## Abstract

**Background:**

Mental health problems are common in the working population and represent a growing concern internationally, with potential impacts on workers, organisations, workplace health and compensation authorities, labour markets and social policies. Workplace interventions that create workplaces supportive of mental health, promote mental health awareness, destigmatise mental illness and support those with mental disorders are likely to improve health and economical outcomes for employees and organisations. Identifying factors associated with successful implementation of these interventions can improve intervention quality and evaluation, and facilitate the uptake and expansion. Therefore, we aim to review research reporting on the implementation of mental health promotion interventions delivered in workplace settings, in order to increase understanding of factors influencing successful delivery.

**Methods and analysis:**

A scoping review will be conducted incorporating a stepwise methodology to identify relevant literature reviews, primary research and grey literature. This review is registered with Research Registry (reviewregistry897). One reviewer will conduct the search to identify English language studies in the following electronic databases from 2008 through to July 1, 2020: Scopus, PROSPERO, Health Technology Assessments, PubMed, Campbell Collaboration, Joanna Briggs Library, PsycINFO, Web of Science Core Collection, CINAHL and Institute of Occupational Safety and Health (IOSH). Reference searching, Google Scholar, Grey Matters, IOSH and expert contacts will be used to identify grey literature. Two reviewers will screen title and abstracts, aiming for 95% agreement, and then independently screen full texts for inclusion. Two reviewers will assess methodological quality of included studies using the Mixed Methods Appraisal Tool and extract and synthesize data in line with the RE-AIM framework, Nielson and Randall’s model of organisational-level interventions and Moore’s sustainability criteria, if the data allows. We will recruit and consult with international experts in the field to ensure engagement, reach and relevance of the main findings.

**Discussion:**

This will be the first systematic scoping review to identify and synthesise evidence of barriers and facilitators to implementing mental health promotion interventions in workplace settings. Our results will inform future evaluation studies and randomised controlled trials and highlight gaps in the evidence base.

**Systematic review registration:**

Research Registry (reviewregistry897)

**Supplementary Information:**

The online version contains supplementary material available at 10.1186/s13643-020-01570-9.

## Background

Mental health problems are common in the working population and represent a growing concern, with potential impacts on workers’ wellbeing, health and discrimination; organisations through lost productivity; workplace health and compensation authorities due to growing job stress-related claims; and social welfare systems owing to increased working age disability pensions for mental disorders [1]. Mental health refers to ‘a state of wellbeing in which the individual realizes his or her own abilities, can cope with normal stresses of life, can work productively and fruitfully, and is able to make a contribution to his or her community’ [2]. Mental health problems therefore include daily worries, stress, burnout and poor wellbeing, as well as mental health conditions such as depression or anxiety [3]. Psychosocial stresses in the workplace, such as job uncertainty, low job control, poor management, harassment and bullying, poor communication and long hours, have been shown to undermine mental wellbeing [4]. A negative working environment may lead to physical and mental health problems, harmful use of substances or alcohol, absenteeism, presenteeism and lost productivity [5]. Although it is acknowledged that mental health problems exist in the workplace, stigma and the social exclusion of people with mental health problems may be leading to under-recognition of such problems and the subsequent low treatment rate of mental health problems [6–8]. Under-treatment has been shown to increase the indirect cost of mental disorders, physical morbidity and mortality [9, 10].

Several studies have evaluated workplace interventions targeting mental wellbeing [11]. Workplace interventions that support mental health and wellbeing have been shown to help reduce sickness absence [12]. In addition, workplaces that promote mental health awareness, destigmatise mental illness and support people with mental disorders are more likely to reduce levels of depression and absenteeism while increasing productivity as well as benefiting from associated economic gains [13]. Improving access to evidence-based interventions for minor stress-related depressive symptoms in occupational sectors associated with high suicide rates, e.g. construction, healthcare and information communication and technology (ICT), is likely to prevent the development of severe depressive disorders and comorbidities, and subsequent suicidal behaviour [13].

Although high-quality evaluations underpin evidence-based interventions (EBI), implementation research can improve the quality of such evaluations and facilitate the uptake and reach of EBIs and other research findings into practice [14]. One effective way to do this is to identify factors that influence the delivery and uptake of interventions during development, feasibility, evaluation and implementation stages [15].

So far, research into specific mechanisms and process factors associated with the successful delivery of mental health promotion interventions in the workplace is limited [16, 17]. This review aims to identify and analyse research on the implementation of workplace mental health promotion interventions; specifically, to understand the barriers and facilitators that influence their delivery in order to provide insights and inform future intervention, evaluation and implementation efforts. This work represents a direct response to recent calls within intervention research to examine the mechanisms through which interventions bring about change and the documentation of contextual and procedural considerations that either facilitate or limit implementation [16, 17].

### Aims and objectives

This review is part of a wider project intending to develop, evaluate and implement a multi-level intervention (Mental Health Promotion and Intervention in Occupational Settings, MENTUPP) [18], which aims to improve mental health and wellbeing in the workplace involving 15 European and Australian partners, with a particular focus on small to medium sized enterprises (SMEs) in three sectors with high prevalence rates of mental health problems and suicidal behaviour, namely ICT, healthcare and construction sectors. More broadly, the purpose of this review is to collate and critically appraise workplace mental health intervention implementation literature to understand how and why certain interventions are more effectively implemented than others and inform MENTUPP and future programmes. The objectives of the review are to:

1. Systematically identify and document research explicitly reporting on the quality of *delivery and implementation* of mental health promotion interventions in workplaces (e.g. reporting the quality of implementation, a process evaluation or realist evaluation) and, if the evidence allows, specifically in ICT, construction and healthcare settings and SMEs.

2. Identify the barriers and facilitators associated with the quality of implementation of mental health promotion interventions in workplace settings and, if the evidence allows, specifically in ICT, construction or healthcare settings and within SMEs, as it relates to the MENTUPP programme of work.

Based on these objectives, our *research questions are:*

i. What is the scope of research with explicit analysis of implementation aspects of mental health promotion interventions in the workplace?
ii. What are the barriers and facilitators to implementing mental health promotion interventions in the workplace?
iii. What are the barriers and facilitators to implementing mental health promotion interventions in SMEs and in the ICT, construction and healthcare sectors?

## Methods/design

### Study design

We will conduct a systematic scoping review using the 6-stage scoping review framework [19, 20] to systematically identify the implementation evidence and factors associated with successful implementation of mental health promotion in workplace settings. Scoping reviews aim to map a broad field of literature and to summarise and disseminate research findings [19, 21], rather than address very focussed questions. This approach is in line with the aims of this review, given the wide range of potential successful and failed interventions, contexts and implementation factors. We will comprehensively explore the relevant research, using iterative methods to develop a rigorous and systematic search of the existing literature [20]. We will recruit and consult with international experts in the field according to both applied organisational and research experience at key stages of the review process and subsequently to ensure engagement, reach and relevance of the process and main findings. The active involvement of people affected by a research topic has been argued to be beneficial to the quality, relevance and impact of research [22, 23], and it enhances the perceived usefulness of systematic review evidence and addresses barriers to the uptake of synthesised research evidence [24, 25].

Our protocol was developed using the Preferred Reporting Items for Systematic Reviews and Meta-Analyses (PRISMA) Protocol checklist (PRISMA-P) [26] (see Additional file 1). The present protocol has been registered within the Research Registry (reviewregistry897). The results of our scoping review will be reported in accordance with PRISMA-ScR [27].

Operationally, the current review will systematically conduct the searches based on the following *definition of key terms*:

*● Implementation*: The results of this review will inform the design of a feasibility and definitive trial of mental health promotion in the workplace. As such, implementation refers to interventions being delivered at feasibility and piloting, evaluation and implementation stages of the Medical Research Council (MRC) framework (15).

*● Mental health promotion* refers to interventions or programmes that aim to treat (intervene to improve mental health), prevent (inhibit the escalation of subclinical symptoms to clinical severity or prevent the onset of mental health problems) and promote (improve mental health by targeting positive components of mental health) mental health and wellbeing [28].

*● Barriers* are defined as any variable or condition that impedes the implementation or delivery of mental health promotion interventions.

*● Facilitators* are defined as any variable or condition that facilitates or improves the implementation or delivery of mental health promotion interventions.

*● Workplace settings* include any organisation operating with paid employees. Therefore, mental health promotion interventions must be delivered through, or be associated with, the workplace. Sector-specific definitions from the European Commission were used [29]. The ICT sector will include telecommunications activities, information technology activities and other information service activities (divisions 61–63); the healthcare sector will include healthcare provided by medical professionals in hospitals or other facilities and residential activities, but not social work activities (divisions 86–87); and the construction sector will include construction of buildings, civil engineering and specialised construction activities (divisions 41–43). Small- to medium-sized enterprises include those employing < 250 employees [30].

### Information sources and search strategy

We will use iterative methods to develop and apply a rigorous and comprehensive search strategy, combining a series of free text terms and Medical Subject Headings (MeSH) terms for key concepts: (a) workplace AND (b) mental health, AND (c) interventions, AND (d) implementation. A preliminary search strategy (see Additional file 2) has been developed for PsycINFO, using established search terms (from Cochrane and other previous search strategies [31–33], peer-reviewed in accordance with PRESS guidelines [34]. Boolean operators will be used to maximise the penetration of terms searched, and appropriate “wild cards” will be employed to account for plurals, variations in databases, and spelling.

We will use a stepwise methodology [35] to identify the highest quality evidence in a systematic way and capture grey literature. Grey literature will be included because it is likely that due to publication bias some unsuccessful interventions have not been published in peer-reviewed journals. A number of contingency plans have been built into the methods to allow an iterative approach to the search and selection of evidence for the review (Additional file 3). We will use established search terms and adapt searches for each of the following major electronic databases outlined below.

In step 1, we will search the following electronic databases for systematic reviews:

●Scopus

● PROSPERO

● Health Technology Assessments

● PubMed

● Campbell Collaboration

● Joanna Briggs Library

● Web of Science Core Collection

In step 2, we will look for primary studies reporting implementation of mental health promotion interventions in the following electronic databases:

● PsychINFO

● Scopus

● PubMed

● Web of Science Core Collection

● CINAHL

● Institute of Occupational Safety and Health (IOSH) research database.

Step 3 will involve supplementary searches involving a thorough review of relevant study references, grey literature and personal contacts using a systematic approach (Additional file 3). This will include searching:

*● Reference searching*: relevant studies included in published guidelines, relevant systematic reviews and listed in the included studies’ reference lists and bibliographies.

● *Grey literature*: Google Scholar (25 pages relevant), Grey Matters and the Institute of Occupational Safety and Health (IOSH) research database.

● *Personal contacts*: we will contact international experts and authors of papers reporting trials (from 2008) on workplace interventions to address mental health promotion.

### Criteria for considering studies for inclusion

#### Overview

The scoping review will address factors associated with successful implementation and therefore focus primarily on feasibility and process studies or realist evaluations. Although we will look at the relation between implementation and effects, the main aim of the review is to identify factors associated with implementation, specifically barriers and facilitators. The focus of this review will be cognisant of outcomes indicating successful implementation, including programme uptake, retention and impact.

#### Study designs

We will include any paper, regardless of study design, using either quantitative, qualitative or mixed-methods, which *explicitly* investigates, reports or discusses, in the title or abstract, any aspect of implementation of specific mental health promotion interventions (i.e. quality of implementation, a process evaluation including rich data or a realist evaluation) delivered in the workplace. This includes literature reviews (systematic reviews, scoping reviews, meta-analyses) and primary research studies published either in the peer-reviewed scientific literature or in the grey literature. We will exclude opinion pieces, commentaries, website discussions, blogs and magazine and newspaper articles.

#### Population

We will include studies with adult participants (aged 16–65) who are in formal employment, including those on sickness absence leave and are expected to return to work.

#### Interventions

Interventions, whose implementation is of interest, are purposefully applied strategies delivered in the workplace, targeting either workers, supervisors, managers, occupational health professionals, owners/executives or entire organisations. Included interventions will aim to (i) help protect mental health by reducing work-related risk factors (e.g. job strain, poor working conditions and job stressors such as job insecurity, psychological harassment (e.g. due to stigma), low social support at work, organisational injustice, and effort-reward imbalance); (ii) promote workplace mental health wellbeing by creating positive aspects of work, and develop employees’ strengths (e.g. satisfaction, wellbeing, psychological capital, positive mental health, resilience and positive organisational attributes such as authentic leadership, supportive workplace culture and workplace social capital); and (iii) respond to mental health problems when they occur (e.g. interventions targeting burnout, stress, anxiety, depression or return to work) [36]. We will exclude studies that evaluate the implementation of general mental health interventions that are not specifically associated with workplace factors or delivered in work contexts (e.g. healthy eating or exercise at home), mental health interventions that are not formally implemented in the workplace (e.g. online work-related mental health interventions freely available online without association to an organisation) and one-off events (e.g. distribution of mental health educational material or one-off information sessions through guest lecturers). Interventions not directly targeting psychological wellbeing or mental health will be included if the primary outcome is related to psychological wellbeing or mental health (e.g. a physical activity programmes delivered in the workplace with a primary outcome for improving mental health). Interventions that target a wide range of health and wellbeing outcomes, e.g. physical activity, obesity, smoking cessation and stress, will be excluded.

#### Outcomes of interest

We will only include studies reporting rich data on any implementation outcomes and will categorise outcomes within our data charting. We anticipate that identified outcomes may include fidelity, reach, dose delivered, dose received, adoption, penetration, feasibility, acceptability, context factors, process factors, sustainability factors, programme theories, theories of change and failure theories. We will exclude studies focusing on only the impact of interventions on disease end points, i.e. which do not evaluate implementation quality.

#### Types of settings

We will include studies conducted in any geographical location, and we will categorise the location based on relevance to Europe and Australia during data charting. The intervention must be delivered in, or in association with, a workplace setting and be implemented in the work schedule, work systems or administrative structures.

#### Language

Studies published in English will be included in steps 1 and 2. Studies published in English, French and German will be included in step 3.

#### Publication date

Studies published in the last 13 years will be included. The World Health Organization’s (WHO) Global Plan of Action on Work’s Health (2008–2017) [37] and the Mental Health Action Plan (2013–2020) [38] highlight the importance of promoting good mental health in the workplace. Furthermore, the field of implementation science is fairly new; therefore, literature published after 2008 is deemed to be most relevant to this review.

### Study selection

Rayyan will be used for the study selection process [39]. Two reviewers will be utilised for a provisional screening of all titles (CP, CL), removing any clearly irrelevant papers. To ensure reliability between reviewers, 15% of the study titles will be reviewed blindly by both reviewers independently, aiming for 95% agreement. Where 95% agreement is not reached, a further 15% will be reviewed by both reviewers independently. Any discrepancy between reviewers will be discussed and, if necessary, will involve a third reviewer to resolve. The remaining study titles will be screened for abstract review by a single reviewer. Two reviewers will then be involved in screening the remaining potential abstracts (CP, CL) and rate them as relevant, irrelevant or unsure. To ensure consistency between reviewers, 15% will be checked independently, and where agreement does not reach 95%, a further 15% will be reviewed by both reviewers. Studies that are ranked as irrelevant will be excluded. We will obtain the full papers for the remaining studies. Two reviewers (CP, CL) will then independently assess each of these against the selection criteria. We will resolve any disagreement through discussion and will involve a third independent reviewer if needed.

### Charting the data

#### Data extraction

We will pilot a data extraction template on the first four included studies and amend as required. We will extract key study details (e.g. study design, country, sample size, sector, intervention characteristics, impact on primary outcome, etc.) and implementation data (e.g. direct quotes, page numbers) will be structured using an adapted version of the RE-AIM framework [40] which has been complemented using selected categories from Nielson and Randall’s model of organisational-level interventions [16] and Moore’s sustainability criteria [41]. To ensure reliability, data from 15% of included papers will be coded by two reviewers (CP and CL) independently. Any ambiguity identified will be resolved through discussion with other members of the review team. Study authors will be contacted via email where data are missing or unclearly reported.

#### Data coding

Data will be coded as follows:

● Stage of intervention development/evaluation will be coded according to the MRC framework (i.e. feasibility, evaluation or implementation) [15].

● Countries will be coded using the World Bank classification [42] to identify countries of relevance to future research, e.g. Europe and Australia.

● Implementation evidence will be mapped using a modified version of the RE-AIM framework [40], which is organised into five categories: reach, effectiveness, adoption, implementation and maintenance. This framework also allows evaluation of implementation at an individual and organisational level.

● Nielson and Randall’s model of organisational-level interventions [16] will supplement the RE-AIM framework for this review allowing for extraction based on the intervention itself, the context in which it was delivered and participants’ mental models.

● Intervention sustainability will be coded using Moore’s definitions of sustainability [41], e.g. continued delivery, behaviour change, evolution/adaptation and continued benefits.

### Quality appraisal

In line with previous systematic and scoping reviews that include mixed methods literature [32, 43], the methodological quality of included studies will be assessed using the Mixed Methods Appraisal Tool (MMAT) [44] for quantitative, qualitative and mixed methods research designs. Each study will receive a methodological rating between 0 and 100 (with 100 being the highest quality), based on the evaluation of study selection bias, study design, data collection methods, sample size, intervention integrity and analysis. Where studies integrate the process evaluation into the study design, the quality of the entire study will be assessed. Methodological quality will be rated by two reviewers (CL and CP). To ensure consistency between reviewers, 15% will be rated independently, and if agreement is reached, one reviewer will rate the remaining papers. Any ambiguity identified will be resolved through discussion with other members of the review team.

### Collating, summarising and reporting

Descriptive characteristics of included studies will be tabulated and brought together using a narrative synthesis. To answer question one, we will summarise the type of evidence relating to the implementation of the interventions in workplace settings. To answer questions two and three, barriers and facilitators will be categorised according to the RE-AIM framework [40], modified using Nielson & Randall’s (2013) model for evaluation organisational-level interventions [16] and Moore’s sustainability criteria [45]. We will present tabulated data by sector and then occupational level (i.e. organisational, managerial, etc.) and intervention type. If the evidence allows, to further answer research question three, we will present tabulated data from included studies focusing specifically on SMEs using the same format. Key findings will be brought together within a narrative synthesis [46, 47].

## Discussion

The aim of this systematic scoping review is to identify research that reports on the feasibility and implementation of mental health promotion interventions that are delivered in workplace settings, and to specifically understand the factors (barriers and facilitators) that influence the successful delivery of mental health promotion interventions in the workplace. This review is part of the MENTUPP project [18] which aims to develop, evaluate and implement mental health promotion interventions for the workplace, particularly in SMEs in the construction, healthcare and ICT sectors. As such, our review will aim to focus on intervention implementation barriers and facilitators in SMEs and in the construction, healthcare and ICT sectors. This work addresses recent calls within intervention research to examine the mechanisms through which interventions bring about change and the documentation of contextual and procedural considerations that either facilitate or limit implementation [16, 17]. Additionally, this timely review responds to international policy regarding mental health in the workplace [8]. In an effort to maintain quality and identify all relevant information, we have presented a rigorous and systematic approach to this scoping review. We have maintained a broad search strategy in order to capture the variety of implementation research that may be available, and we will consult with stakeholders to ensure the main findings are useful and relevant. The results of this review will identify barriers and facilitators to implementation of mental health promotion interventions in the workplace and inform future pilot and definitive RCTs within the MENTUPP project [18]. This will help inform future interventions, and the evaluation and implementation efforts of such interventions, which will subsequently improve outcomes for employees and organisations through improved mental wellbeing; reduced symptoms of depression, anxiety and stress; and reduced presenteeism and absenteeism. In addition, this review will contribute to implementation science related to workplace mental health promotion.

## Supplementary Information

**Additional file 1.** PRISMA-P checklist.

**Additional file 2.** Draft Search Strategy.

**Additional file 3.** Outline of the step-wise review methodology.

## Data Availability

All data generated or analysed during this study will be included in the published scoping review article and will be available by request to the corresponding author.

## Abbreviations

ICT: Information and communication technology
IOSH: Institution of occupational safety and health
MRC: Medical research council
MeSH: Medical subject heading
MMAT: Mixed methods appraisal tool
PRISMA: Preferred reporting items for systematic reviews and meta-analyses
PRISMA-P: Preferred reporting items for systematic reviews and meta-analyses
PRISMA-ScR: Preferred reporting items for systematic reviews and meta-analyses extension for scoping reviews
RCT: Randomized controlled trial
RE-AIM: Reach, effectiveness, adoption, implementation and maintenance
SMEs: Small-to-medium sized enterprises
WHO: World health Organization
