# Supplementary material for: Evidence for implementation of interventions to promote mental health in the workplace: a systematic scoping review protocol
Source: Syst Rev. 2021 Jan 28;10:41. doi: 10.1186/s13643-020-01570-9 (PMC7844910; doi:10.1186/s13643-020-01570-9)
Supplement: Supplementary file 3 — Additional file 3. Outline of the step-wise review methodology. [file 13643_2020_1570_MOESM3_ESM.docx]

**Additional Files 3: Outline of step-wise review methodology**

| **Step 1: Search for systematic reviews** | |  | |  |  | |  | |  |
| --- | --- | --- | --- | --- | --- | --- | --- | --- | --- |
| **Reference screening** | 2 reviewers will screen by title, then by abstract, then by full texts to select studies for inclusion. | **Step 2: Search for primary studies with particular attention paid to gaps in evidence identified in step 1** | | |  | |  | |  |
|  |  | **Study types included** | Qualitative studies; surveys; trials with process evaluations | | | **Step 3: Search for grey literature with particular attention paid to gaps in evidence identified in steps 1 and 2** | | | |
| **Quality control check** | Quality control check of 15% of title topics, then abstracts, until 95% agreement reached between 2 reviewers. Two reviewers will screen full texts independently. | **Reference screening** | 2 reviewers will screen by title topic, then by abstract and then full text to select studies for inclusion. | | | **Internet search** | | Google Scholar (25 pages relevant), Grey Matters and IOSH. | |
| **Identify gaps in the evidence** | Selected references mapped to the 3 work sectors to identify gaps. | **Quality control check** | Quality control check of 15% of title topics, then abstracts, until 95% agreement reached between 2 reviewers. Two reviewers will review full texts independently. | | | **Snowball** | | Scan reference lists in literature retrieved through Steps 1 and 2 for further publications. | |
| **Team consensus on studies for inclusion** | Final agreement on studies for inclusion.  Are there gaps in evidence? If so, progress to step 2. | **Identifying gaps in the evidence** | Selected references mapped the 3 work sectors | | | **Call for evidence** | | Stakeholders, will be consulted to identify unpublished reports that may inform the review and to seek published and peer reviewed studies missed. | |
|  |  | **Team consensus on studies selected for inclusion** | Final agreement on studies selected for inclusion.  Are there gaps in the evidence? If so, progress to step 3 | | | **Identifying gaps**  **Team consensus on evidence** | | Selected references mapped to sectors to identify gaps.  Final agreement on studies selected for inclusion.  Are there gaps in evidence? If so, clearly document. | |
